# The PCNA inhibitor AOH1996 suppresses cancer stemness and enhances anti-PD1 immunotherapy in squamous cell carcinoma

**DOI:** 10.1186/s13287-025-04607-9

**Published:** 2025-09-29

**Authors:** Yujia Wang, Zhen Qin, Yiwen Chen, Baoxin Gu, Lingfei Jia

**Affiliations:** 1 Department of Oral and Maxillofacial Surgery, Peking University School and Hospital of Stomatology, Beijing, 100081 China; 2 National Center for Stomatology & National Clinical Research Center for Oral Diseases & National Engineering Research Center of Oral Biomaterials and Digital Medical Devices, Beijing, 100081 China; 3 Beijing Advanced Center of Cellular Homeostasis and Aging-Related Diseases, Institute of Advanced Clinical Medicine, Peking University, Beijing, 100081 China

**Keywords:** Head and neck squamous cell carcinoma (HNSCC), AOH1996, PCNA, Cancer stem cell (CSC), Immunotherapy

## Abstract

**Background:**

Proliferating cell nuclear antigen (PCNA), a well-documented anticancer target, is critical for DNA synthesis, replication, and repair. AOH1996, a small-molecule PCNA inhibitor, is currently undergoing clinical trials for the treatment of advanced solid tumors. However, the therapeutic effect of AOH1996 on head and neck squamous cell carcinoma (HNSCC) remains unclear.

**Methods:**

The effects of AOH1996 on HNSCC biological behaviors and cancer stemness were tested in HNSCC cells and nude mice. The combination treatment of AOH1996 and anti-PD1 was performed in a 4-nitroquinoline N-oxide (4NQO)-induced HNSCC mouse model. RNA sequencing, Western Blotting, immunofluorescence staining, comet assays, and qRT‒PCR were conducted for mechanistic studies.

**Results:**

Our results showed that AOH1996 effectively inhibited HNSCC proliferation and invasion both in vitro and in vivo. AOH1996 suppressed HNSCC stemness, development, and metastasis. Moreover, AOH1996 altered the tumor immune microenvironment into an inflamed state with increased CD8^+^ T-cell infiltration, rendering it a favorable partner for combination therapy with immune checkpoint inhibitors. Mechanistically, AOH1996 induced cellular DNA damage, suppressed cancer stemness through the upregulation of p-TBK1, and promoted the secretion of CD8^+^ T-cell-recruiting chemokines by stimulating IRF3-mediated transcription.

**Conclusions:**

Taken together, our results demonstrated that AOH1996 suppressed tumor growth, eliminated cancer stem cells (CSCs), and synergistically enhanced the efficacy of anti-PD1 immunotherapy in HNSCC.

**Supplementary Information:**

The online version contains supplementary material available at 10.1186/s13287-025-04607-9.

## Background

Head and neck squamous cell carcinoma (HNSCC) is the most common malignant tumor of the head and neck [1]. HNSCC is derived mainly from the mucosal epithelium of the oral cavity, pharynx, and larynx [2]. HNSCC is highly aggressive and frequently metastasizes to cervical lymph nodes, resulting in a poor prognosis [2]. The conventional treatments include surgery, chemotherapy, radiotherapy, and multimodal approaches [2–4]. However, the 5-year survival rate for HNSCC, which ranges from 35 to 70% [5, 6], has not improved in recent years, attributed to HNSCC metastasis, recurrence, and drug resistance [7, 8].

For metastatic and recurrent HNSCC, the Food and Drug Administration has approved immune checkpoint inhibitor (ICI) therapy, including pembrolizumab and nivolumab, which target the programmed cell death protein 1 (PD1) receptor [2, 9, 10]. However, only approximately 20% of patients with HNSCC achieve a durable response to ICI treatment, indicating that most patients with HNSCC are resistant to PD1 blockade-based immunotherapy [11–13]. Thus, more effective therapeutic targets and strategies for HNSCC are needed.

Proliferating cell nuclear antigen (PCNA) reflects the status of cell proliferation and is located mostly in the nuclei of normal proliferating cells and tumor cells [14]. In the form of a homotrimeric ring structure, PCNA encircles DNA and is essential for DNA synthesis, replication, and repair [15–17]. PCNA is highly expressed in HNSCC tumors compared with adjacent normal epithelium and serves as a cell proliferation marker in HNSCC [18, 19]. Thus, PCNA is a potential target for cancer treatment [20]. Recently, AOH1996, a small-molecule PCNA inhibitor that targets transcription replication conflict, has been reported [21]. AOH1996 is lethal to malignant tumors but not normal cells and inhibits cancer-associated PCNA [22]. AOH1996 suppresses tumor growth as a monotherapy or in combination with chemotherapy without discernible side effects [21]. AOH1996 is orally active, metabolically stable, and currently in clinical trials for the treatment of advanced solid tumors. However, the therapeutic effect of AOH1996 on HNSCC remains unclear.

Cancer stem cells (CSCs) are a subpopulation of cancer cells capable of self-renewal, infinite proliferation, and multiple differentiation potential [23–25]. CSCs are closely associated with HNSCC initiation, development, metastasis, recurrence, and drug resistance [26, 27], and serve as promising targets for HNSCC treatment [28–30].

In this study, we aimed to evaluate the therapeutic potential of AOH1996, with a particular focus on its effects on cancer stemness and the tumor immune microenvironment, and to elucidate the underlying mechanisms involved. These findings may provide a better understanding of the therapeutic potential of AOH1996 in HNSCC and promote the development of more effective treatment strategies targeting PCNA and its associated pathways in cancer.

## Methods

### Cell culture and treatment

The human HNSCC cell lines CAL27 [31] and SCC15 [32] (American Type Culture Collection, ATCC, Manassas, VA, USA), both HPV-negative, were cultured in Dulbecco’s modified Eagle’s medium (DMEM) supplemented with 10% fetal bovine serum (FBS; Vazyme, Nanjing, China; Cat# F101-01) at 37 °C in a humidified atmosphere containing 5% CO_2_. The culture medium contained 1% penicillin‒streptomycin solution (Aoqing Biotechnology Co., Ltd., Beijing, China; Cat# AQ512). AOH1996 (Selleck, Shanghai, China; Cat# E159601) was dissolved in dimethyl sulfoxide (DMSO; Aoqing Biotechnology Co., Ltd.; Cat# AQ62650). After reaching 70%‒80% confluence, HNSCC cells were treated with 1 µM DMSO or AOH1996.

### Cell counting Kit-8 (CCK-8) assay

The proliferation of HNSCC cells was evaluated using a CCK-8 assay (Dojindo, Shanghai, China). The cells treated with AOH1996 were added to 96-well plates (2 × 10^3^ cells/well). Ten microliters of CCK-8 reagent was added to each well at 0, 24, 48, and 72 h and incubated at 37 °C for 1 h before detection. The absorbance at 450 nm was measured using a microplate spectrophotometer (Bio-Tek Instruments Inc., Winooski, VT, USA). The IC50 and EC50 values of the HNSCC cells were calculated.

### Transwell assay

Transwell chambers with 8 μm pore-size membranes coated with Matrigel (Corning Inc., NY, USA; Cat# 3422) were placed in 24-well plates. HNSCC cells were resuspended in 200 µL of serum-free DMEM (1 × 10^5^ cells) and added to the upper chamber. DMEM containing 20% FBS (600 µL) was added to the lower chamber. After 24 h of incubation at 37 °C, the cells on the upper side of the membranes were removed using a cotton swab. Invaded cells were fixed with 4% paraformaldehyde and stained with 0.1% crystal violet aqueous solution (Solarbio, Beijing, China; Cat# G1064). The membranes were then washed with phosphate-buffered saline (PBS). Images were acquired using an optical microscope (Olympus, Tokyo, Japan). The number of invaded cells was randomly counted from three fields and analyzed using ImageJ (NIH, Bethesda, MD, USA).

### Terminal deoxynucleotidyl transferase nick-end-labeling (TUNEL) assay

A TUNEL Apoptosis Assay Kit (Solarbio; Cat# T2130) was used to detect apoptotic cells following the manufacturer’s instructions. Briefly, the cells were incubated with TUNEL working solution for 1 h at 37 °C after AOH1996 treatment for 24 h. The cells were fixed with 4% paraformaldehyde for 20 min and then stained with 4′,6-diamidino-2-phenylindole (DAPI; Solarbio; Cat# C0065). Images were acquired using a fluorescence microscope.

### CSC isolation and flow cytometry assay

After AOH1996 treatment, an ALDEFLUOR Kit (STEMCELL Technologies, Vancouver, Canada; Cat# 01700) was used to isolate CSCs from HNSCC cells expressing high levels of aldehyde dehydrogenase (ALDH^high^). DEAB is a specific inhibitor of ALDH and is used as an ALDH^low^ control for background fluorescence. A fluorescence-activated cell sorting (FACS) assay was used to separate ALDH^high^ and ALDH^low^ subpopulations in HNSCC cells. To isolate CSCs from human HNSCC patient-derived xenograft (PDX) model #1, a human Tumor Dissociation Kit (Miltenyi Biotec, Bergisch Gladbach, Germany; Cat# 130-095-929) was used to digest the HNSCC PDX tumors into single cells. The single-cell suspension was incubated with an EpCAM antibody (Miltenyi Biotec; Cat# 130-111-116) to isolate EpCAM^+^ tumor cells. An ALDEFLUOR Kit was then used to separate ALDH^high^ and ALDH^low^ subpopulations in EpCAM^+^ tumor cells. The flow cytometry results were analyzed using FlowJo software (Becton, Dickinson & Company, New Jersey, USA).

### Tumorsphere formation assay

Ultralow attachment 6-well plates (Corning Inc.; Cat# 3471) were used to analyze tumorsphere formation. FACS-isolated HNSCC cells treated with AOH1996 were plated. Serum-free DMEM/F12 (Thermo Fisher Scientific, Shanghai, China; Cat# 8122659) supplemented with 1% N2 (Thermo Fisher Scientific; Cat# 17502048) and 1% B27 supplement (Thermo Fisher Scientific; Cat# 17504044) was used to culture the cells. A total of 20 ng/mL recombinant human epidermal growth factor (EGF; R&D Systems, Minneapolis, MN, USA; Cat# 236-EG-01 M) and 10 ng/mL human basic fibroblast growth factor (FGF; R&D Systems; Cat# 233-FB/CF) [33] were added to the medium. Microscopy images of tumorspheres with diameters greater than 70 μm were acquired, and the tumorsphere volumes were evaluated after 14 days.

### Western blot

HNSCC cells were lysed using radioimmunoprecipitation assay (RIPA) buffer (Solarbio; Cat# R0010) containing phenylmethylsulfonyl fluoride (PMSF; Beyotime, Shanghai, China; Cat# ST506). Protein samples were separated on 10% SDS polyacrylamide gels and transferred to polyvinylidene difluoride (PVDF) membranes. The membranes were blocked with 5% nonfat milk for 1 h and then incubated with primary antibodies at 4 °C overnight. The primary antibodies used included the following: anti-BMI1 (1:1000; Cell Signaling Technology, Shanghai, China; Cat# 6964), anti-SOX2 (1:1000; Cell Signaling Technology; Cat# 14962), anti-ALDH1 (1:1000; Cell Signaling Technology; Cat# 54135), anti-MYC (1:1000; Cell Signaling Technology; Cat# 18583), anti-phosphorylated H2A.X (anti-p-H2A.X; 1:1000; Cell Signaling Technology; Cat# 9718), anti-p-STING (1:1000; Cell Signaling Technology; Cat# 19781), anti-STING (1:1000; Cell Signaling Technology; Cat# 13647), anti-p-TBK1 (1:1000; Cell Signaling Technology; Cat# 5483), anti-TBK1 (1:1000; Cell Signaling Technology; Cat# 3504), anti-p-IRF3 (1:1000; Cell Signaling Technology; Cat# 29047), and anti-IRF3 (1:1000; Cell Signaling Technology; Cat# 4302). GAPDH (1:1000; Cell Signaling Technology; Cat# 5174) was used as the internal control. The membranes were then incubated with secondary horseradish peroxidase (HRP)-labeled goat anti-rabbit or goat anti-mouse antibodies (Beyotime; Cat# A0208 or #A0216) at room temperature for 1 h. Immunoreactive protein bands were visualized using NcmECL Ultra (NCM Biotech, Suzhou, China; Cat# P10100) reagent.

### RNA-seq and pathway enrichment analysis

Total RNA was isolated from SCC15 cells using TRIzol Reagent (Thermo Fisher Scientific; Cat# 15596026). The quality of total RNA was evaluated using an Agilent 2100 Bioanalyzer (Agilent Technologies, Santa Clara, CA, USA). An Illumina TruSeq RNA sample preparation kit (Illumina, San Diego, CA, USA) was used for RNA-sequencing library construction [34]. An Illumina HiSeq 2500 sequencer was used to sequence single-ended RNA. Kyoto Encyclopedia of Genes and Genomes (KEGG) pathway analysis was performed using the DAVID Bioinformatics platform (https://davidbioinformatics.nih.gov/tools.jsp). The RNA-seq data were deposited in the Gene Expression Omnibus (GEO) database of the National Center for Biotechnology Information under accession number GSE289770.

### Comet assay

A Comet SCGE Assay Kit (Enzo, NY, USA; Cat# ADI-900-166) was used for single-cell gel electrophoresis [35]. After HNSCC cells were treated with AOH1996 for 24 h, the cells (1 × 10^5^ cells/ml) were mixed with molten LM agarose at a 1:10 (v/v) ratio. Prewarmed slides were loaded with 75 µl aliquots and placed at 4 °C in the dark for 10 min. The slides were immersed in prechilled lysis solution for 1 h, followed by prechilled alkaline solution for 40 min and TBE buffer for 10 min. Slides in TBE buffer were subjected to horizontal electrophoresis at 25 V for 20 min. Slides were immersed in 70% ethanol for 5 min, dried at room temperature, and stained with CYGREEN dye solution for 30 min. Images were acquired using a fluorescence microscopy. At least 50 cells per sample were analyzed using the CASP analysis tool Version 1.2.2 (CASPlab, Wroclaw, Poland).

### Cytosolic DsDNA staining

After AOH1996 treatment for 24 h, HNSCC cells were stained with PicoGreen (Thermo Fisher Scientific; Cat# P11496) for 1 h and MitoTracker (Thermo Fisher Scientific; Cat# M7512) for 30 min. The cells were fixed with 4% paraformaldehyde for 15 min, and the nuclei were stained with DAPI. Images were acquired using a confocal laser scanning microscope (Olympus FV3000, Tokyo, Japan).

### Quantitative reverse transcription polymerase chain reaction (qRT‒PCR)

Total RNA was extracted from HNSCC cells using TRIzol Reagent (Thermo Fisher Scientific; Cat# 15596026), and then 500 ng of RNA was reverse transcribed to cDNA using a reverse transcription kit (Takara, Beijing, China; Cat# RR036A). A SYBR Green Kit (Roche, Basel, Switzerland; Cat# 04913914001) was used to quantify the cDNA by quantitative real-time PCR, which was conducted at 95 °C for 10 min, followed by 40 cycles of 95 °C for 15 s and 60 °C for 1 min using an ABI Prism 7500 real-time PCR System (Applied Biosystems, Foster City, CA, USA). The primers used for qRT**‒**PCR are listed in Table S1.

### In vivo HNSCC tumor growth, ELDA, patient-derived xenografts (PDXs), and the orthotopic model

Female BALB/c nude mice (6–8 weeks old) were purchased from SPF Biotechnology Co., Ltd (Beijing, China). All the animal studies strictly conformed to the regulations of the Institutional Animal Care and Use Committee of Peking University Health Science Center. In this study, the mice were euthanized by cervical dislocation after isoflurane anesthesia. The work has been reported in line with the ARRIVE guidelines 2.0.

For the subcutaneous tumor models, twelve mice were randomly divided into two groups (*n* = 6/group). SCC15 cells were suspended in a 1:1 mixture of PBS and Matrigel matrix (Corning Inc., Bedford, MA, USA; Cat# 354234), and then subcutaneously injected (5 × 10^6^ cells/mouse) into the flanks of the mice. 5% AOH1996 was dissolved in 95% corn oil. One week after injection, vehicle or AOH1996 (50 mg/kg) was used to treat the mice orally twice a week for three weeks. Tumor size was measured weekly. After four weeks, the tumors were harvested, photographed, and weighed (g).

For the extreme limiting dilution assay (ELDA), ALDH^high^ cells were obtained from SCC15 cells using FACS. The FACS-sorted CSCs were suspended in a 1:1 mixture of PBS and Matrigel matrix, and then subcutaneously injected into the flanks of the mice. One week after injection, vehicle or AOH1996 (50 mg/kg) was used to treat the mice orally twice a week for three weeks. The results were analyzed using ELDA software: https://bioinf.wehi.edu.au/software/elda/index.html.

For the HNSCC PDX and orthotopic models, CSCs were obtained from the HNSCC PDX #1 model using FACS with EpCAM^+^ ALDH^high^ markers, as previously described [36]. FACS-sorted HNSCC CSCs were injected orthotopically into the tongues of mice (1 × 10^6^ cells/mouse). One week after injection, thirty mice were randomly divided into two groups (*n* = 15/group) and treated with vehicle or AOH1996 (50 mg/kg) orally twice a week for three weeks.

After the mice were sacrificed, the tongues and cervical lymph nodes were collected. The tumor volume (mm^3^) was calculated as V = 1/2 × D × d^2^, where D is the longer diameter and d is the shorter diameter.

### HNSCC 4-nitroquinoline 1-oxide (4NQO) mouse model, treatment and histology

For 4NQO-induced HNSCC, 6–8-week-old *Bmi1*^*CreER*^, *Rosa*^*tdTomato*^ mice were given 50 µg/ml 4NQO (Santa Cruz, CA, USA; Cat# 256815) in the drinking water for 16 weeks, followed by normal drinking water for 6 weeks. For lineage tracing, tamoxifen (9 mg/40 g weight, Sigma–Aldrich, St. Louis, MO, USA; Cat# T5648), which labels BMI1^+^ CSCs, was intraperitoneally injected into the mice 2 days before sacrifice.

For HNSCC treatment, after 22 weeks, the mice were randomly divided into four groups and treated as follows: (1) control vehicle; (2) AOH1996 (50 mg/kg, twice a week); (3) anti-PD1 (BioXcell, Lebanon, NH, USA; Cat# BE0146, 200 µg/mouse, twice a week); and (4) AOH1996 combined with anti-PD1, which lasted four weeks. To assess the tolerability of anti-PD1, AOH1996, and combination treatments in mice, the heart, liver, lung, kidney, and spleen were collected for histopathological analysis, and H&E-stained images were captured. The blood of the mice was collected for routine blood tests and biochemical indicator analysis. HNSCC tumor growth and lymph node metastasis were evaluated as previously described [36]. Tongues and lymph nodes were collected after sacrifice, and tongue lesion areas were measured.

For histological analysis and immunostaining, longitudinally cut tongues (dorsal/ventral) and lymph nodes were sectioned into 4-µm-thick tissues, fixed with 4% paraformaldehyde, and paraffin-embedded overnight. Tongue sections were stained with hematoxylin and eosin (H&E). The number of squamous cell carcinomas (SCCs) was counted, and SCC areas were measured as previously described [36]. The HNSCC invasion score was based on the following criteria. Grade 1: normal or epithelial dysplasia; Grade 2: distinct invasion, unclear basement membrane, and drop or diffuse infiltration into the superficial muscle layer; Grade 3: basement layer loss and extensive invasion into the deep muscle layer.

### Immunostaining

For immunohistochemistry (IHC), after antigen repair, the lymph node sections were incubated with the primary antibody anti-pan-cytokeratin (anti-PCK; 1:200; Santa Cruz Biotechnology, Shanghai, China; Cat# sc-8018) at 4 °C overnight. Anti-PCK can be used to specifically test epithelial tumor cells in lymph nodes. The tongue sections were incubated with the primary antibody anti-CD8 (1:200; Cell Signaling Technology; Cat# 98941) at 4 °C overnight. The sections were then incubated with goat anti-mouse IgG polymer (ZSGB-BIO; Cat# PV-6002) or goat anti-rabbit IgG polymer (ZSGB-BIO; Cat# PV-6001) secondary antibodies for 1 h at room temperature. The percentages of lymph node metastasis, metastatic areas, and CD8^+^ T cells were quantified.

For cell immunofluorescence staining, HNSCC cells were fixed with 4% paraformaldehyde for 15 min after AOH1996 treatment at room temperature. The cells were incubated with the primary antibody anti-p-H2A.X (1:100; Cell Signaling Technology; Cat# 9718) at 4 °C overnight, followed by incubation with a secondary antibody conjugated with the fluorescent marker Cy3 (Jackson ImmunoResearch Laboratories, West Grove, PA, USA). The nuclei were stained with DAPI.

All immunostaining images were acquired using a fluorescence microscope.

### Statistical analysis

Statistical analyses were performed using GraphPad Prism 10.4.1 for Windows (GraphPad Software Inc., La Jolla, CA, USA). In vitro experiments were performed at least three times, and in vivo experiments were performed at least twice. The data are expressed as the mean ± standard deviation (SD) or standard error of the mean (SEM) [28]. Differences between two groups were analyzed using Student’s t test. Differences among multiple groups were analyzed by one-way analysis of variance (ANOVA) [33]. A two-tailed value of *P* < 0.05 was considered statistically significant.

## Results

### AOH1996 inhibits cell proliferation and invasion while promoting apoptosis in HNSCC cells

**Fig. 1:**
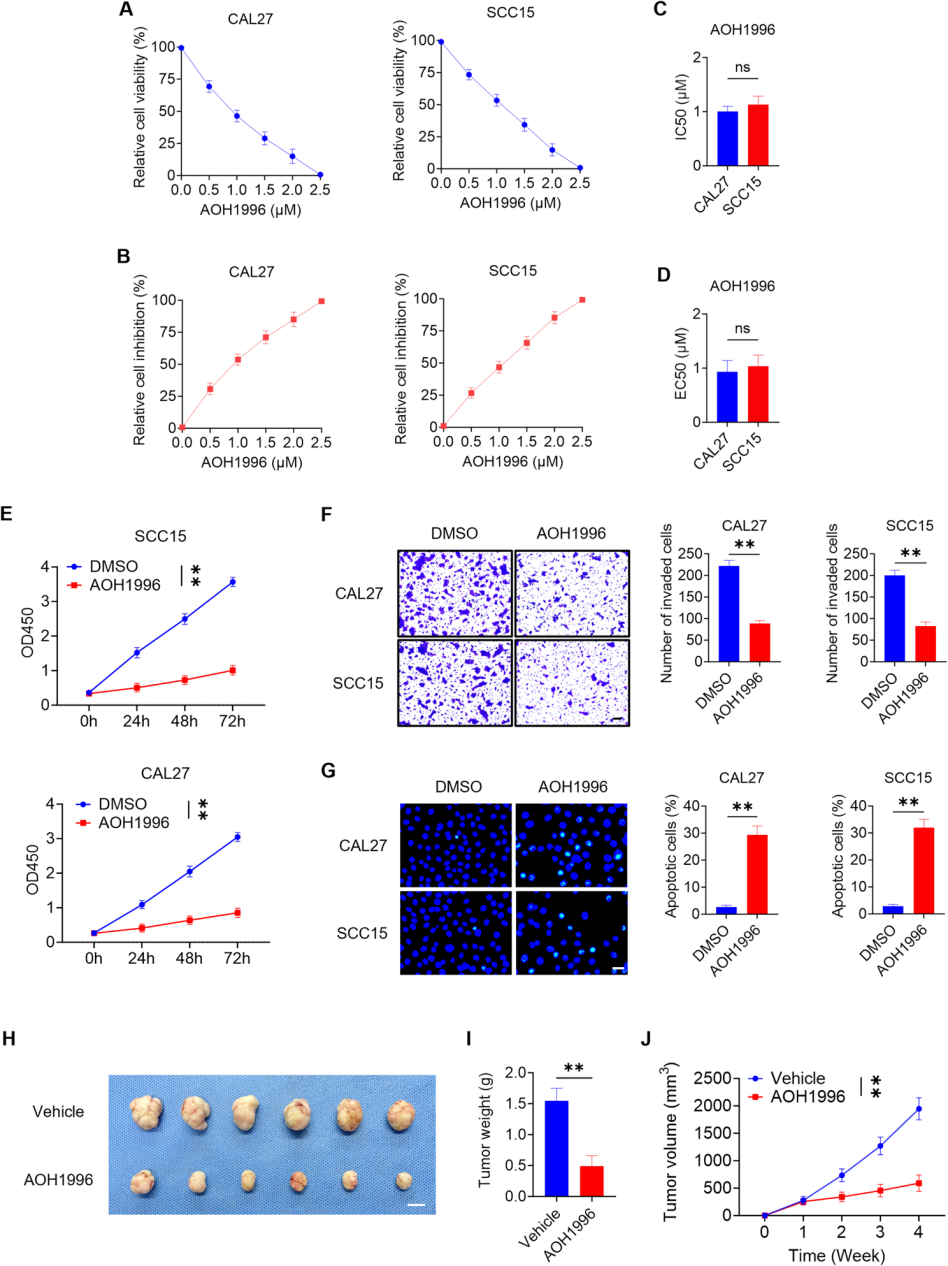
AOH1996 inhibits HNSCC cell proliferation and invasion, promotes apoptosis, and suppresses tumor growth (**A-B**) Relative cell viability and relative cell inhibition of CAL27 and SCC15 cells treated with AOH1996 determined by CCK-8 analysis. Values and error bars represent the means ± SDs. (**C-D**) The IC50 and EC50 values of CAL27 and SCC15 cells treated with AOH1996 determined by CCK-8 analysis. Values and error bars represent the means ± SDs with an unpaired Student’s t test. ns, not significant. (**E**) Effects of AOH1996 on the proliferation of CAL27 and SCC15 cells after 0–72 h, as determined in a CCK-8 assay. Optical density (OD) 450 values and error bars represent the means ± SDs with an unpaired Student’s t test. **P < 0.01. (**F**) Numbers of invaded CAL27 and SCC15 cells treated with AOH1996 determined by the Transwell assay. Values and error bars represent the means ± SDs with an unpaired Student’s t test. Scale bar = 100 μm. **P < 0.01. (**G**) Apoptosis rates of CAL27 and SCC15 cells treated with AOH1996. Values and error bars represent the means ± SDs with an unpaired Student’s t test. Scale bar = 20 μm. **P < 0.01. (**H**) Images of subcutaneous tumors formed by SCC15 cells treated with AOH1996 in nude mice. Scale bar = 1 cm. (**I**) Weights of subcutaneous tumors formed by SCC15 cells treated with AOH1996 in nude mice. Values and error bars represent the means ± SDs with an unpaired Student’s t test (*n* = 6). **P < 0.01. (**J**) Volume growth curves of subcutaneous tumors formed by SCC15 cells treated with AOH1996 in nude mice for four weeks. Values and error bars represent the means ± SDs with an unpaired Student’s t test (*n* = 6). **P < 0.01

To investigate the therapeutic effect of AOH1996 on HNSCC, two HNSCC cell lines, CAL27 and SCC15, were treated with AOH1996. AOH1996 reduced the relative cell viability of CAL27 and SCC15 cells (Fig. 1A). Moreover, AOH1996 increased the relative cell inhibition of CAL27 and SCC15 cells (Fig. 1B). Based on the IC50 and EC50 values of AOH1996, the concentration of 1 µM AOH1996 was selected for further study (Fig. 1C-D). A CCK-8 assay revealed that AOH1996 inhibited the proliferation of CAL27 and SCC15 cells (Fig. 1E). A Matrigel invasion assay revealed that AOH1996 inhibited the invasion of CAL27 and SCC15 cells (Fig. 1F). Moreover, AOH1996 increased the percentages of apoptotic CAL27 and SCC15 cells (Fig. 1G). To assess whether AOH1996 could inhibit tumor growth in vivo, AOH1996 was given to nude mice with SCC15 cell-derived tumors. Compared with the control treatment, AOH1996 treatment reduced the weight and volume of HNSCC tumors (Fig. 1H-J).

### AOH1996 inhibits the cancer stemness signature of HNSCC

**Fig. 2:**
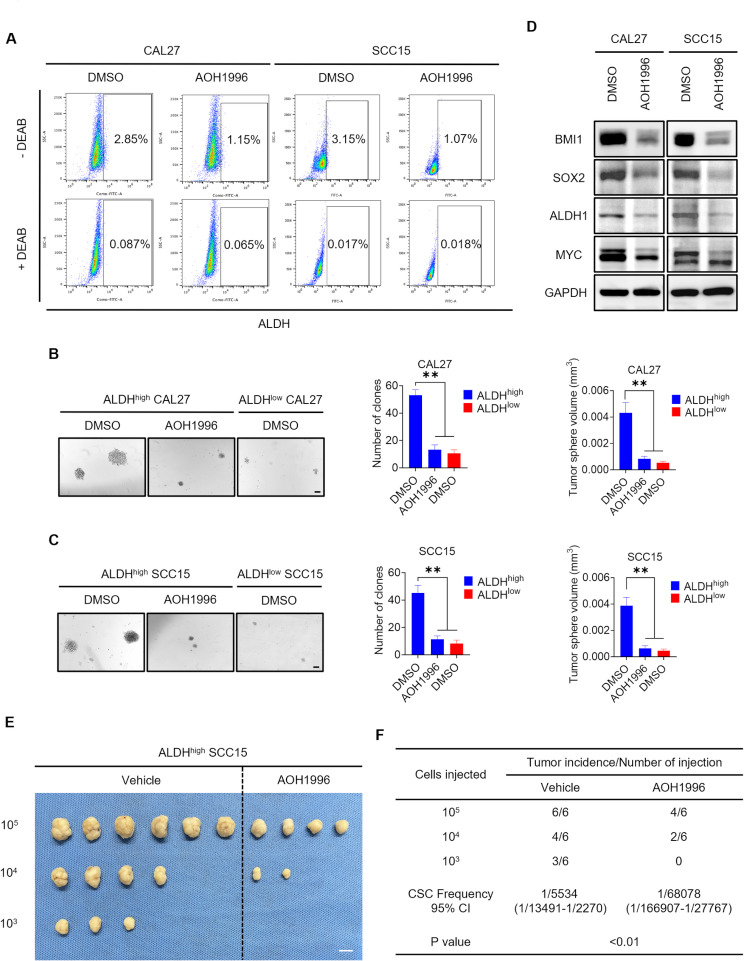
AOH1996 inhibits the cancer stemness of HNSCC (**A**) Representative fluorescence-activated cell sorting (FACS) images of ALDH^high^ CAL27 and SCC15 cells treated with AOH1996. N-Diethylaminoazobenzene (DEAB) was used as a control for background fluorescence. The percentage of ALDH^high^ cells is indicated in the gate. (**B-C**) Representative images and quantification of tumorsphere formation in ALDH^high^ and ALDH^low^ CAL27 and SCC15 cells treated with AOH1996. Values and error bars represent the means ± SDs with an unpaired Student’s t test. Scale bar = 100 μm. **P < 0.01. (**D**) Western blot showing the protein levels of BMI1, SOX2, ALDH1, and MYC in CAL27 and SCC15 cells treated with AOH1996. GAPDH was used as the internal control. The full-length blots are presented in Supplementary Material 1. (**E-F**) Extreme limiting dilution analysis (ELDA) of ALDH^high^ CSC-like SCC15 cells treated with AOH1996 in vivo (*n* = 6). Scale bar = 1 cm. The table shows the frequency of tumor formation at each cell dose injected. The data were analyzed using ELAD software

We further investigated whether AOH1996 could inhibit cancer stemness in HNSCC. Aldehyde dehydrogenase (ALDH) is a specific marker on the surface of CSCs [23, 37]. Using an ALDEFLUOR Kit, we sorted ALDH^low^ nonstem tumor cells and ALDH^high^ CSC-like cells with the specific ALDH inhibitor diethylaminobenzaldehyde (DEAB) as a control. Fluorescence-activated cell sorting (FACS) analysis revealed that the percentages of ALDH^high^ CSC-like cells among CAL27 and SCC15 cells after AOH1996 treatment were significantly lower than those in the control group (Fig. 2A). AOH1996 treatment inhibited the tumorsphere formation of CAL27 and SCC15 ALDH^high^ CSC-like cells, reducing the number and volume of tumorspheres (Fig. 2B-C). BMI1, SOX2, ALDH1, and MYC are classical markers of CSCs [30, 38, 39]. Western blot analysis revealed that BMI1, SOX2, ALDH1, and MYC protein levels were significantly reduced in CAL27 and SCC15 cells treated with AOH1996 (Fig. 2D). Moreover, an in vivo extreme limiting dilution assay (ELDA) indicated that AOH1996 treatment reduced the tumor incidence/injection ratio and inhibited the tumorigenic potential of ALDH^high^ SCC15 cells (Fig. 2E-F). These results demonstrated that AOH1996 inhibited the cancer stemness of HNSCC.

### AOH1996 inhibits the progression and metastasis of CSCs cultured from patient-derived xenograft (PDX) models

**Fig. 3:**
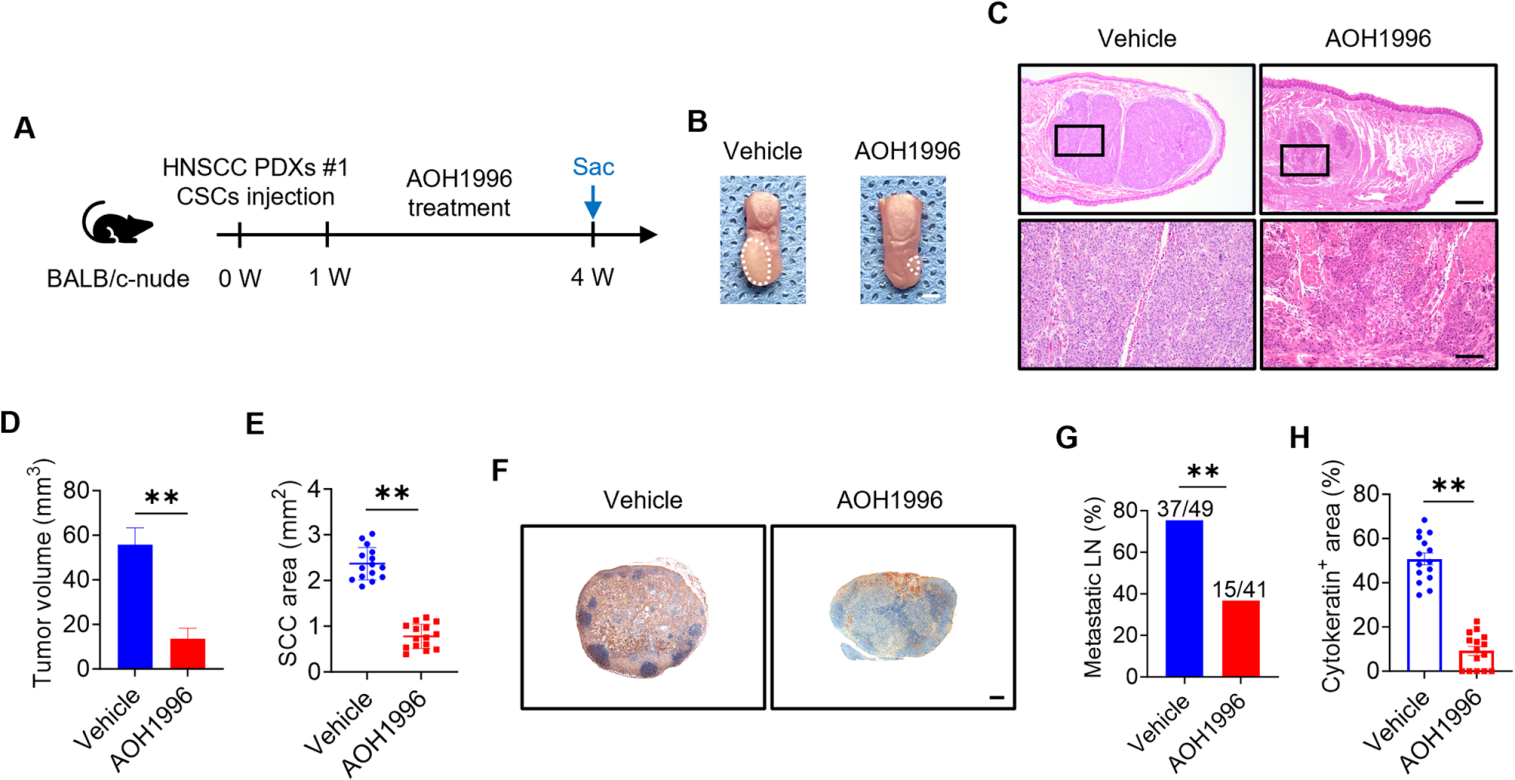
AOH1996 inhibits the tumorigenic potential and lymph node metastasis of HNSCC CSCs (**A**) Schematic diagram showing the timeline of BALB/c-nude mice injected with HNSCC CSCs cultured from patient-derived xenografts (PDXs) #1, AOH1996 treatment, and sacrifice (Sac). (**B**) Representative images of orthotopic tongue tumors from nude mice treated with vehicle or AOH1996. The white dashed circles mark the tumor areas. Scale bar = 2 mm. (**C**) Representative hematoxylin and eosin (H&E) staining images of orthotopic tongue tumors from nude mice treated with vehicle or AOH1996. Scale bar = 500 μm. Magnified images are shown in the lower panels. Scale bar = 100 μm. (**D**) Quantification of orthotopic tongue tumor volume in nude mice treated with vehicle or AOH1996 as indicated. Values and error bars represent the means ± SDs with an unpaired Student’s t test (*n* = 15). **P < 0.01. (**E**) Quantification of the squamous cell carcinoma (SCC) area in nude mice treated with vehicle or AOH1996 as indicated. Values and error bars represent the means ± SDs with an unpaired Student’s t test (*n* = 15). **P < 0.01. (**F**) Representative images of metastatic cells in the cervical lymph nodes from nude mice treated with vehicle or AOH1996 and immunostained with anti-pan-cytokeratin (anti-PCK). Scale bar = 200 μm. (**G**) Percentages of metastatic lymph nodes from nude mice treated with vehicle or AOH1996. Metastatic lymph nodes (LN) numbers are indicated in the histogram. The result represents the average of two independent experiments according to the chi-square test. **P < 0.01. (**H**) Quantification of the metastatic area in cervical lymph nodes from nude mice treated with vehicle or AOH1996. Values and error bars represent the means ± SEMs from two independent experiments; unpaired Student’s t tests were performed. **P < 0.01

PDX models and human cancers share similar genetic landscapes, and PDXs have been widely used as preclinical models in cancer studies [40]. Here, we used a human HNSCC PDX model to evaluate the therapeutic efficacy of AOH1996. CSCs were isolated from the HNSCC PDX #1 model using FACS with EpCAM^+^ ALDH^high^ markers, as previously described [36]. The FACS-sorted HNSCC CSCs from the PDX model were injected orthotopically into the tongues of the mice (Fig. 3A). The results showed that AOH1996 treatment suppressed the growth of HNSCC tumors (Fig. 3B-C). Consistently, AOH1996 treatment significantly reduced the tumor volume and area (Fig. 3D-E). Metastasis is a complex process in which cancer cells migrate and invade from the primary tumor to distant sites [41]. CSCs are associated with this process because of their ability to self-renew and differentiate into various cell types within the tumor [37, 42]. Immunohistochemistry (IHC) staining with an anti-pan-cytokeratin (anti-PCK) antibody indicated that, compared with vehicle treatment, AOH1996 treatment inhibited the cervical lymph node metastasis of CSCs (Fig. 3F-H). Thus, AOH1996 treatment effectively inhibited the tumorigenic potential and prevented the progression and metastasis of HNSCC CSCs derived from the PDX model.

### AOH1996 eliminates CSCs and enhances the response to anti-PD1 immunotherapy in HNSCC

**Fig. 4:**
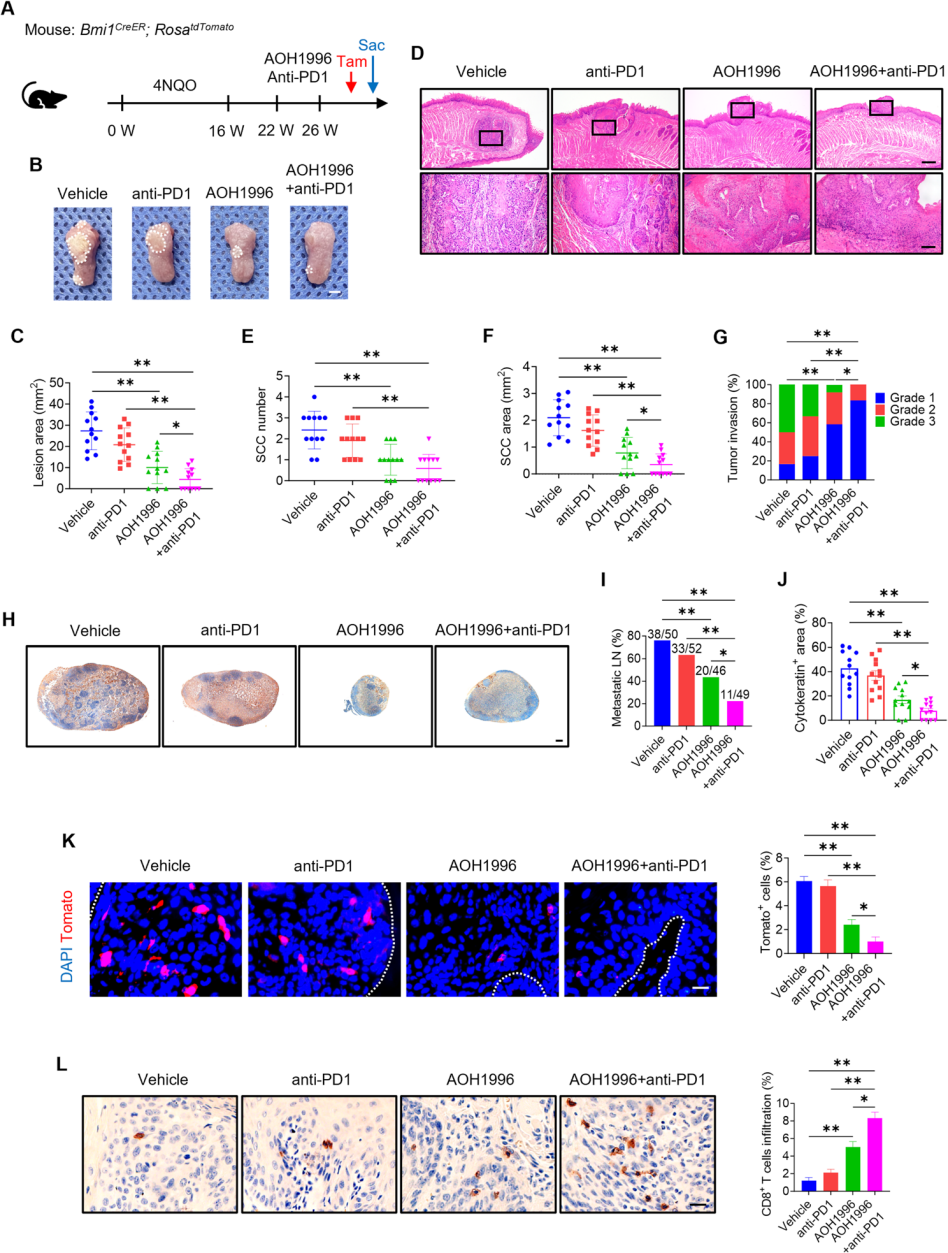
AOH1996 enhances the therapeutic effect of anti-PD1 immunotherapy in HNSCC (**A**) Schematic diagram showing the timeline of *Bmi1*^*CreER*^, *Rosa*^*tdTomato*^ mouse lineage tracing of primary HNSCC, treatment, tamoxifen (Tam) injection, and sacrifice (Sac). (**B**) Representative images of HNSCC orthotopic tongue lesions in the different groups. The white dashed circles mark the lesion areas. Scale bar = 2 mm. (**C**) Quantification of the HNSCC lesion area. Values and error bars represent the means ± SDs (*n* = 12) according to one-way analysis of variance (ANOVA). *P < 0.05 and **P < 0.01. (**D**) Representative H&E staining images of HNSCC orthotopic tongue tumors. Scale bar = 500 μm. Magnified images are shown in the lower panels. Scale bar = 100 μm. (**E-F**) Quantification of the SCC number and area. Values and error bars represent the means ± SDs of one-way ANOVA (*n* = 12). *P < 0.05 and **P < 0.01. (**G**) Quantification of the HNSCC invasion grade by the Cochran–Armitage test. *P < 0.05 and **P < 0.01. (**H**) Representative anti-PCK immunostaining images of metastatic cells in cervical lymph nodes. Scale bar = 200 μm. (**I**) Percentage of metastatic lymph nodes. The number of metastatic lymph nodes is indicated in the histogram by the chi-square test. *P < 0.05 and **P < 0.01. (**J**) Quantification of the metastatic area in the cervical lymph nodes. Values and error bars represent the means ± SEMs, as determined by one-way ANOVA. *P < 0.05 and **P < 0.01. (**K**) Representative immunofluorescence images and quantification of Tomato^+^ BMI1^+^ CSCs in HNSCC. The white dashed lines mark the tumor-stromal boundary. Scale bar = 20 μm. Values and error bars represent the means ± SDs of one-way ANOVA. *P < 0.05 and **P < 0.01. (**L**) Representative immunostaining images and quantification of CD8^+^ T cells infiltration in HNSCC. Scale bar = 20 μm. Values and error bars represent the means ± SDs of one-way ANOVA. *P < 0.05 and **P < 0.01

In our previous study, we successfully established a *Bmi1*^*CreER*^, *Rosa*^*tdTomato*^ mouse model of HNSCC using 4-nitroquinoline 1-oxide (4NQO) induction. This model leverages the specific labeling of Bmi1^+^ tumor cells (CSCs) with tdTomato fluorescence, enabling precise tracking and analysis of CSC dynamics in vivo [28]. The 4NQO-induced carcinogenesis model closely mimics the histopathological features of human HNSCC, providing a robust approach for investigating whether AOH1996 treatment could eliminate CSCs and synergize with anti-PD1 immunotherapy (Fig. 4A).

Compared with either anti-PD1 monotherapy or AOH1996 monotherapy, combination treatment with AOH1996 and anti-PD1 significantly reduced the tongue lesion area (Fig. 4B-C). Compared with either treatment alone, histological analysis demonstrated that the combination treatment significantly reduced the number, area, and invasion of HNSCC cells (Fig. 4D-G). The combination therapy effectively suppressed cervical lymph node metastasis in HNSCC, as evidenced by anti-PCK immunostaining (Fig. 4H-J).

In vivo lineage tracing revealed that AOH1996 reduced Bmi1^+^ CSCs in HNSCC, whereas the combination treatment of AOH1996 and anti-PD1 led to increased elimination of Bmi1^+^ CSCs (Fig. 4K). Moreover, we investigated the impact of the combination treatment on the tumor immune microenvironment. Immunostaining revealed that the combination treatment significantly increased the infiltration of CD8^+^ T cells into the tumor microenvironment (Fig. 4L). These results demonstrated that the combination treatment of AOH1996 and anti-PD1 not only inhibited the CSCs and metastasis of HNSCC but also enhanced tumor cell-intrinsic immune responses, highlighting its potential as a synergistic therapeutic strategy for HNSCC.

**Fig. 5:**
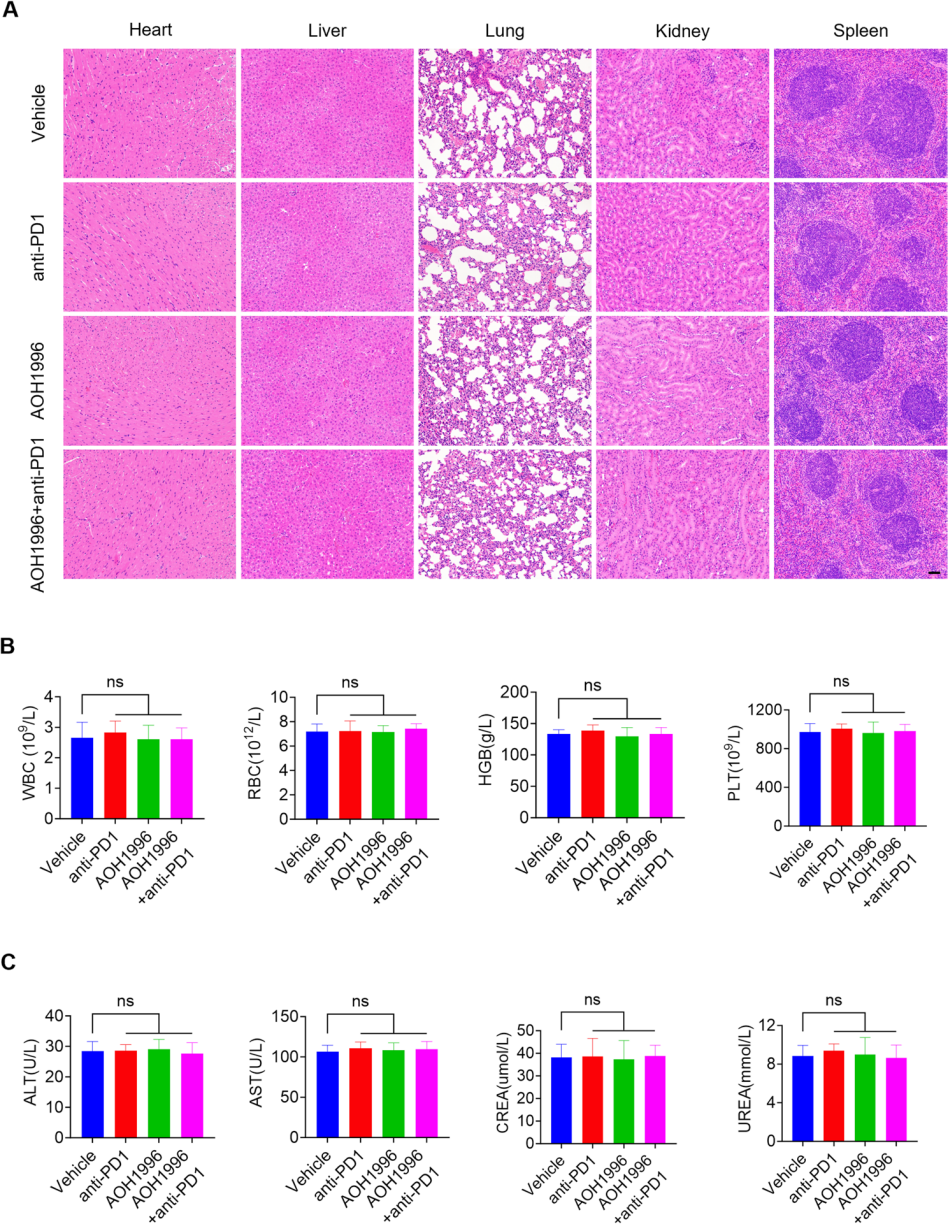
Systematic effect of AOH1996 combined with anti-PD1 treatment in HNSCC in vivo (**A**) Representative hematoxylin and eosin (H&E) staining images of heart, liver, lung, kidney, and spleen from C57BL/6J mice treated with vehicle, AOH1996, anti-PD1, or AOH1996 combined with anti-PD1. Scale bar = 200 μm. (**B**) The diagram shows the results of routine blood analysis in different treatment groups, which included tests of white blood cell (WBC), red blood cell (RBC), hemoglobin (HGB), and platelet (PLT) levels in the mice. Values and error bars represent the means ± SDs of one-way ANOVA. ns, not significant. (**C**) Diagram showing the blood biochemical indicators in different treatment groups, which include the test of alanine aminotransferase (ALT), aspartate aminotransferase (AST), creatinine (CREA), and urea levels in the mice. Values and error bars represent the means ± SDs of one-way ANOVA. ns, not significant

To evaluate the drug safety of the anti-PD1, AOH1996, and the combination treatment of AOH1996 and anti-PD1, we conducted histopathological and hematological analyses in C57BL/6J mice. Histopathological examination revealed no significant morphological alterations in major organs, including the heart, liver, lung, kidney, and spleen (Fig. 5A). Routine blood tests, including assessments of white blood cell (WBC), red blood cell (RBC), hemoglobin (HGB), and platelet (PLT) levels, revealed no notable abnormalities after anti-PD1, AOH1996, or combination treatment (Fig. 5B). Additionally, blood biochemical analyses, in which alanine aminotransferase (ALT), aspartate aminotransferase (AST), creatinine (CREA), and urea levels were measured, revealed no significant changes, confirming the absence of systemic toxicity (Fig. 5C). Collectively, these results demonstrated that anti-PD1, AOH1996, and combination treatment were well tolerated in vivo, supporting the safety and potential clinical applicability of this therapeutic strategy.

### AOH1996 induces cellular DNA damage, activates the cGAS–STING signaling pathway, inhibits cancer stemness, and stimulates antitumor immune responses in HNSCC

**Fig. 6:**
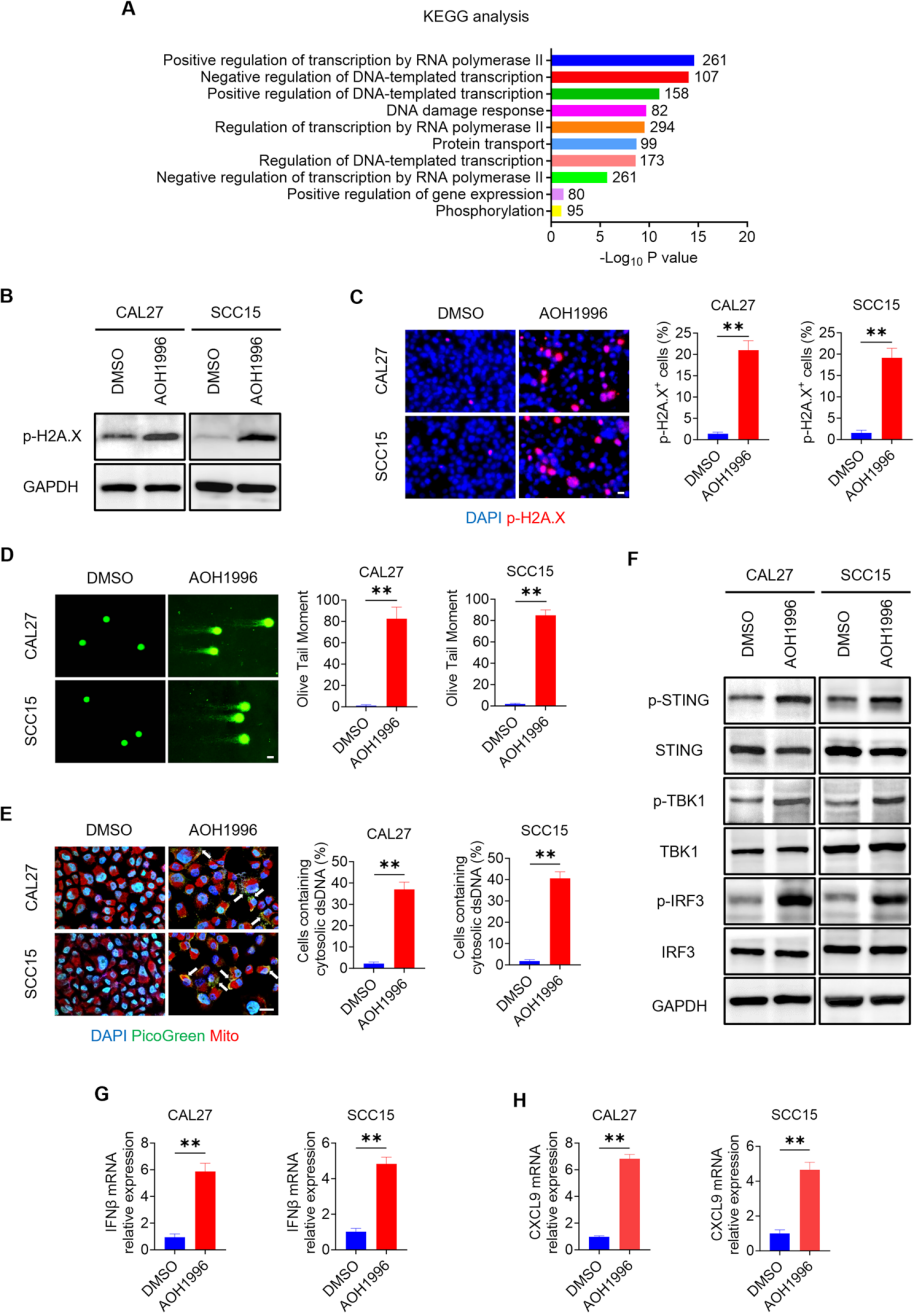
AOH1996 induces cellular DNA damage, activates the cGAS-STING signaling pathway, and stimulates antitumor immune responses (**A**) Histogram showing the top 10 enriched KEGG pathways in SCC15 cells treated with AOH1996 according to RNA sequencing. Fold change > 1.5. Y-axis: categories of the KEGG pathway; X-axis: the statistical significance of the enrichment. The number of genes in each KEGG pathway category is listed on the right. (**B**) Western blot showing the protein levels of p-H2A.X in CAL27 and SCC15 cells treated with AOH1996. The full-length blots are presented in Supplementary Material 1. (**C**) Immunofluorescence staining and quantification of p-H2A.X (red) in CAL27 and SCC15 cells treated with AOH1996. The nuclei were stained with DAPI (blue). Values and error bars represent the means ± SDs with an unpaired Student’s t test. Scale bar = 20 μm. **P < 0.01. (**D**) Representative images and quantification of AOH1996-treated CAL27 and SCC15 cells by the DNA comet assay. Values and error bars represent the means ± SDs with an unpaired Student’s t test. Scale bar = 20 μm. **P < 0.01. (**E**) Representative images and quantification showing the accumulation of cytosolic DNA in CAL27 and SCC15 cells treated with AOH1996. The nuclei, double-stranded DNAs (dsDNAs), and mitochondrial DNAs (mtDNAs) were stained with DAPI (blue), PicoGreen (green), and MitoTracker (red), respectively. The white arrows indicate cytosolic dsDNA. Values and error bars represent the means ± SDs with an unpaired Student’s t test. Scale bar = 20 μm. **P < 0.01. (**F**) Western blot showing the protein levels of p-STING, STING, p-TBK1, TBK1, p-IRF3, and IRF3 in CAL27 and SCC15 cells treated with AOH1996. The full-length blots are presented in Supplementary Material 1. (G-H) qRT‒PCR analysis showing the relative expression levels of IFNβ and CXCL9 mRNAs in CAL27 and SCC15 cells treated with AOH1996. Values and error bars represent the means ± SDs with an unpaired Student’s t test. **P < 0.01

To investigate the mechanisms underlying the therapeutic potential of AOH1996 in HNSCC, RNA sequencing (RNA-seq) was performed on HNSCC cells. Comprehensive transcriptome alterations were analyzed in SCC15 cells after treatment with AOH1996. KEGG pathway analysis suggested that AOH1996 treatment modulated the regulation of transcription by RNA polymerase II, DNA-templated transcription, and DNA damage response signaling pathways (Fig. 6A). Given that PCNA is pivotal in DNA synthesis, replication, and repair, previous studies have shown that AOH1996 can induce DNA double-strand breaks [21]. We focused the subsequent experiments on the DNA damage signaling pathway.

Phosphorylated H2A.X (p-H2A.X) has been used as a DNA damage marker in previous studies [43, 44]. Western blot analysis revealed that AOH1996 increased p-H2A.X protein levels in HNSCC cells (Fig. 6B). Consistently, immunofluorescence staining of the p-H2A.X marker indicated that AOH1996 treatment caused the accumulation of DNA damage in CAL27 and SCC15 cells (Fig. 6C). Nuclear DNA damage in single eukaryotic cells can be evaluated in a comet assay [45]. Damaged DNA fragments separated from intact cellular DNA are shown as comet tails under a fluorescence microscope. The results revealed that AOH1996 treatment increased the comet tail moments of CAL27 and SCC15 cells, suggesting that AOH1996 induced DNA damage in HNSCC cells (Fig. 6D). To investigate whether increased nuclear DNA damage could cause the accumulation of cytosolic DNA, CAL27 and SCC15 cells after AOH1996 treatment were stained with PicoGreen and MitoTracker. PicoGreen is a specific double-stranded DNA (dsDNA) marker that can also stain mitochondrial DNA (mtDNA) [46]. MitoTracker was used to stain the mtDNA to avoid visual interference when the cytosolic dsDNA was quantified. Confocal microscopy images revealed the increased cytosolic DNA accumulation in HNSCC cells treated with AOH1996 (Fig. 6E).

The cyclic GMP–AMP synthase-stimulator of interferon genes (cGAS–STING) signaling pathway can be activated by accumulated cytosolic dsDNA, inducing the phosphorylation of STING, TANK binding kinase 1 (TBK1) and interferon regulatory factor 3 (IRF3). Phosphorylated IRF3 regulates the expression of IFN [47–49]. Western blot analysis revealed that AOH1996 increased the phosphorylated STING (p-STING), p-TBK1, and p-IRF3 protein levels in CAL27 and SCC15 cells (Fig. 6F). The total protein levels of STING, TBK1, and IRF-3 remained unchanged. Thus, AOH1996 activated the cGAS–STING signaling pathway in HNSCC. A previous study showed that increased expression of p-TBK1 inhibited the proliferation and maintenance of CSCs in cancer [50]. Our results indicated that AOH1996 inhibited cancer stemness by increasing the protein level of p-TBK1.

qRT–PCR analysis revealed that AOH1996 increased the relative expression of IFNβ mRNA in CAL27 and SCC15 cells (Fig. 6G). The expression of C-X-C motif chemokine ligand (CXCL) is regulated by IFN [51]. qRT–PCR analysis revealed that AOH1996 increased the relative expression of the CD8^+^ T-cell-recruiting chemokine CXCL9 mRNA in CAL27 and SCC15 cells (Fig. 6H). Thus, AOH1996 stimulated antitumor immune responses in HNSCC.

## Discussion

In recent years, targeted therapies that focus on specific molecules have been widely applied in the treatment of various cancers, which potentiate precise therapeutic strategies [52]. The identification and validation of biomarkers has driven the advancement of targeted therapy, improving the outcomes and prognosis of patients with HNSCC [53]. Previous studies have shown that PCNA is essential for cell proliferation and maintenance of genomic stability [14, 54]. PCNA is closely associated with cancer initiation and progression and serves as a target in anticancer therapy [54]. PCNA reflects the rapid proliferation of HNSCC and is a biomarker for carcinogenesis [18, 19]. A novel small-molecule PCNA inhibitor, AOH1996, has shown great therapeutic potential in many cancers, including non-small cell lung cancer, colon cancer, and breast cancer [21]. Compared with other PCNA inhibitors, T2AA, PCNA-I1, and AOH1160 [22], AOH1996 selectively kills cancer cells but not normal cells [21]. Here, we investigated the therapeutic effect of AOH1996 on HNSCC.

**Fig. 7:**
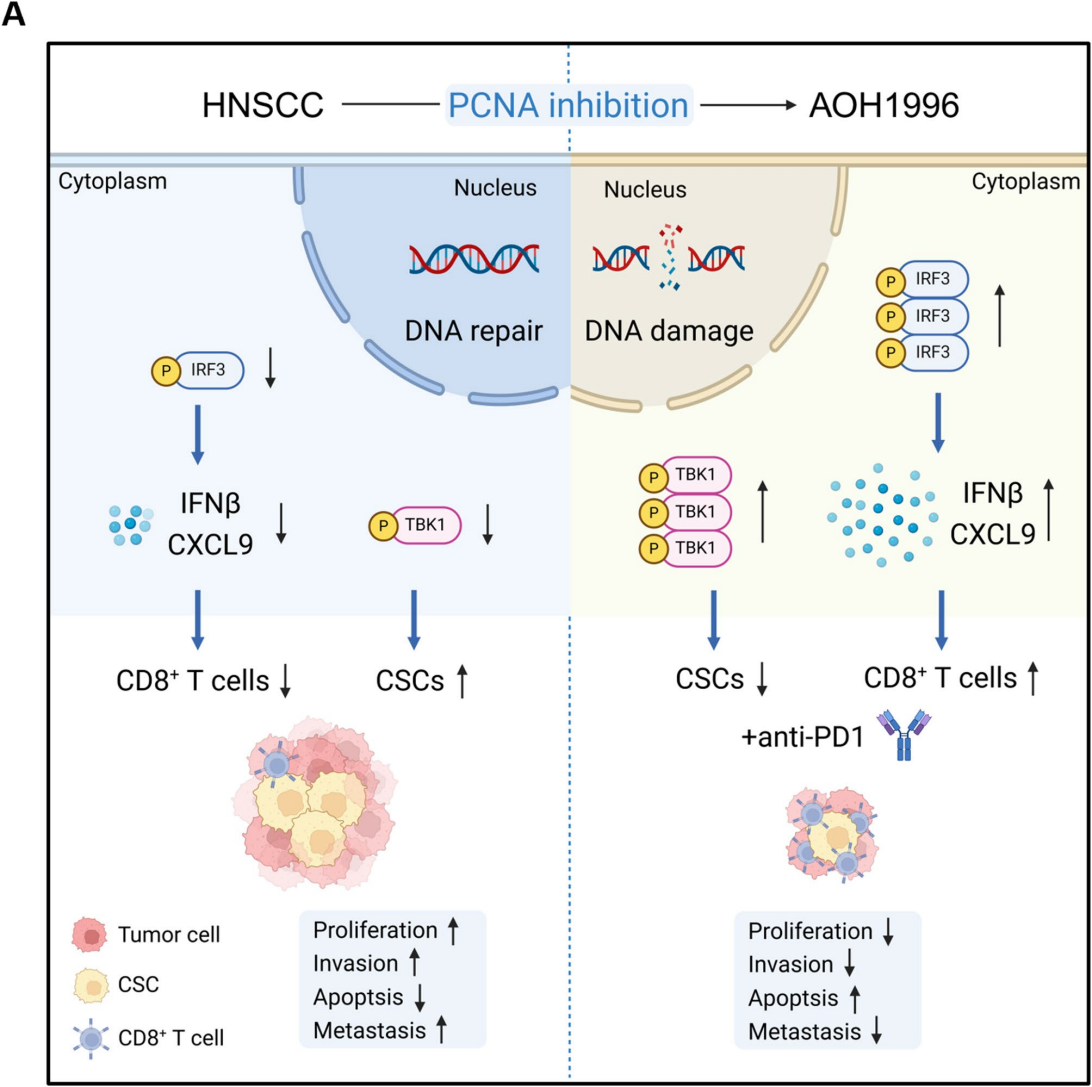
Mechanism of AOH1996 treatment in HNSCC (**A**) Schematic diagram (created with Biorender.com) illustrating the mechanism by which AOH1996 combined with anti-PD1 treatment eliminates CSCs and enhances the efficacy of anti-PD1 immunotherapy in HNSCC

In this study, our results demonstrated that AOH1996 inhibited the biological behaviors of HNSCC both in vitro and in vivo. AOH1996 suppressed HNSCC stemness, development, and metastasis. Moreover, AOH1996 eliminated CSCs, increased CD8^+^ T-cell infiltration, and enhanced the efficacy of anti-PD1 immunotherapy. Mechanistically, AOH1996 induced cellular DNA damage, activated the cGAS–STING signaling pathway, suppressed cancer stemness by upregulating p-TBK1 expression, and promoted CD8^+^ T-cell-recruiting chemokines by stimulating IRF3-mediated transcription (Fig. 7A).

The therapeutic effect of AOH1996 on regulating HNSCC cancer stemness remains unclear. A previous study demonstrated that PCNA is essential for maintaining cancer stemness through Y211 phosphorylation [14]. Loss of Y211 phosphorylation in PCNA impairs cancer stemness, showing reduced ALDH activity and inhibited tumorsphere formation. In contrast, cells with intact Y211 phosphorylation maintain cancer stemness, showing increased ALDH activity and enhanced tumorsphere formation [14]. Thus, we speculated that PCNA inhibition could impair cancer stemness in HNSCC. We further explored whether AOH1996 could inhibit CSCs in HNSCC. Here, we used HNSCC PDX CSC-injected nude mice to explore the therapeutic effect of AOH1996 in vivo. PDX models can reflect the original characteristics of tumor progression in patients with cancer and are genetically similar to human cancers, providing a promising model for clinical translation in cancer treatment [55, 56]. Our results showed that AOH1996 eliminated and targeted CSCs in HNSCC. Moreover, AOH1996 suppressed HNSCC development and metastasis, suggesting the possibility of the clinical application of AOH1996 in patients with HNSCC.

Cancer stemness and immune evasion are important characteristics of HNSCC initiation, development, and metastasis [57, 58]. The interaction between CSCs and the tumor microenvironment enables CSCs to evade immune surveillance in HNSCC [26, 33]. The tumor microenvironment in HNSCC is immunosuppressive with low levels of lymphocyte infiltration [59]. Here, we investigated the effect of AOH1996 on the tumor microenvironment of HNSCC. Immune checkpoint inhibitor therapy has been approved for treating metastatic and recurrent HNSCC. However, most patients with HNSCC are resistant to PD1 blockade-based immunotherapy [11–13]. CSCs can create immunosuppressive niches in the tumor microenvironment and are associated with resistance to PD1 blockade-based immunotherapy [42, 51]. Previous studies have found that CSCs induce immunosuppression through high expression of PD-L1, TGF-β, and the recruitment of regulatory T cells (Tregs) [60]. Thus, the AOH1996 treatment, which inhibits CSCs in HNSCC, may improve the efficacy of immunotherapy.

The 4NQO-induced HNSCC *Bmi1*^*CreER*^, *Rosa*^*tdTomato*^ mouse model enables CSC lineage tracing in vivo [28]. We used this model to investigate the effect of AOH1996 on CSCs in HNSCC. Our results suggested that AOH1996 eliminated CSCs and enhanced the therapeutic effect of anti-PD1 immunotherapy, suppressing the development and metastasis of HNSCC. Both the AOH1996 treatment alone and the combination treatment with anti-PD1 increased the recruitment of CD8^+^ T cells in the tumor microenvironment of HNSCC. The increased infiltration of CD8^+^ T lymphocytes can directly identify, target, and destroy cancer cells in the HNSCC tumor microenvironment, which is related to a favorable prognosis [59, 61]. Thus, AOH1996 targets CSCs in HNSCC and improves the efficacy of anti-PD1 immunotherapy. Taken together, AOH1996 prevented the immune evasion of CSCs, activated the tumor immune response, and inhibited HNSCC progression and metastasis. Moreover, combination treatment with AOH1996 and anti-PD1 exhibited therapeutic effects on HNSCC without causing discernible toxicity or side effects in vivo.

Mechanistically, AOH1996 can cause DNA double-strand breaks and genome instability [21]. Previous studies have shown that the DNA damage response activates innate immunity through the cGAS–STING pathway in cancers [62–65]. In this study, we explored the underlying mechanism of AOH1996 in HNSCC by bioinformatic analysis. The results showed that AOH1996 induced HNSCC cellular DNA damage. Moreover, AOH1996 increased the expression levels of p-STING, p-TBK1, and p-IRF3 in HNSCC, activating the cGAS–STING signaling pathway. A previous study revealed that in glioblastoma cancer, TLR4 overexpression increases the phosphorylation of TBK1, and increasing p-TBK1 inhibits the expression of the CSC markers SOX2, OCT4, and NANOG [50]. Thus, p-TBK1 upregulation inhibits CSC proliferation and maintenance [50]. Our results indicated that AOH1996 eliminated CSCs by upregulating p-TBK1 expression in HNSCC. Moreover, IFN regulates the expression of CXCL chemokines [51]. Our results showed that AOH1996 increased the expression of IFNβ and induced the recruitment of the CD8^+^ T-cell-recruiting chemokine CXCL9 by stimulating IRF3-mediated transcription. Thus, AOH1996 stimulated antitumor immune responses of HNSCC cells by inducing DNA damage and activating the cGAS–STING signaling pathway.

However, there are limitations in this study. The translational potential of AOH1966 is constrained by its unknown effects in human HNSCC samples or patient-derived immune microenvironments. Future studies should further validate the therapeutic efficacy of AOH1996 in human HNSCC samples. The molecular mechanism by which AOH1996 modulates PCNA in HNSCC CSCs requires further investigation. Moreover, how AOH1996 modulates immune checkpoints such as PD-L1 and Tregs also needs to be explored.

## Conclusions

Our study demonstrated that the PCNA inhibitor AOH1996 suppressed HNSCC biological behaviors, impaired cancer stemness, prevented development and metastasis, enhanced the effect of anti-PD1 immunotherapy, promoted cellular DNA damage, and stimulated antitumor immune responses. This study provides a novel therapeutic strategy for HNSCC.

## Supplementary Information

Below is the link to the electronic supplementary material.

Supplementary Material 1

Supplementary Material 2

## Data Availability

The data supporting this study are available from the corresponding author upon reasonable request. The RNA-seq data were deposited in the GEO database of the NCBI under accession number GSE289770.

## Abbreviations

PCNA: Proliferating cell nuclear antigen
HNSCC: Head and neck squamous cell carcinoma
CSCs: Cancer stem cells
ICI: Immune checkpoint inhibitors
PD1: Programmed cell death protein 1
DMEM: Dulbecco’s modified Eagle’s medium
FBS: Fetal bovine serum
DMSO: Dimethyl sulfoxide
CCK-8: Cell counting kit-8
DAPI: 4′,6-diamidino-2-phenylindole
FACS: Fluorescence-activated cell sorting
PDXs: Patient-derived xenografts
EGF: Epidermal growth factor
FGF: Fibroblast growth factor
RIPA: Radioimmunoprecipitation assay
PMSF: Phenylmethylsulfonyl fluoride
PVDF: Polyvinylidene difluoride
HPR: Horseradish peroxidase
ELDA: Extreme limiting dilution assay
4NQO: 4-nitroquinoline 1-oxide
H&E: Hematoxylin and eosin
SCC: Squamous cell carcinoma
IHC: Immunohistochemistry
PCK: Pan-cytokeratin
GEO: Gene Expression Omnibus
qRT–PCR: Quantitative reverse transcription polymerase chain reaction
SD: Standard deviation
SEM: Standard error of the mean
ANOVA: One-way analysis of variance
ALDH: Aldehyde dehydrogenase
WBC: White blood cells
RBC: Red blood cells
HGB: Hemoglobin
PLT: Platelet
ALT: Alanine aminotransferase
AST: Aspartate aminotransferase
CREA: Creatinine
RNA-seq: RNA-sequencing
KEGG: Kyoto Encyclopedia of Genes and Genomes
dsDNA: Double-stranded DNA
mtDNA: Mitochondrial DNA
TBK1: TANK-binding kinase 1
IRF3: Interferon regulatory factor 3
CXCL: C-X-C motif chemokine ligand
