# Supplementary material for: The PCNA inhibitor AOH1996 suppresses cancer stemness and enhances anti-PD1 immunotherapy in squamous cell carcinoma
Source: Stem Cell Res Ther. 2025 Sep 29;16:523. doi: 10.1186/s13287-025-04607-9 (PMC12482506; doi:10.1186/s13287-025-04607-9)
Supplement: Supplementary file 1 — Supplementary Material 1 [file 13287_2025_4607_MOESM1_ESM.pdf]

Table S1. The sequences of qRT-PCR primers in this study.

Target genes	Forward (5'-3')	Reverse (5'-3')
IFNβ	GTCACTGTGCCTGGACCATAG	GTTTCGGAGGTAACCTGTAAGTC
CXCL9	CCAGTAGTGAGAAAGGGTCGC	AGGGCTTGGGGCAAATTGTT
GAPDH	CTGGGCTACACTGAGCACC	AAGTGGTCGTTGAGGGCAATG
