# Supplementary figures and images for: The PCNA inhibitor AOH1996 suppresses cancer stemness and enhances anti-PD1 immunotherapy in squamous cell carcinoma
Source: Stem Cell Res Ther. 2025 Sep 29;16:523. doi: 10.1186/s13287-025-04607-9 (PMC12482506; doi:10.1186/s13287-025-04607-9)

Figure 2D

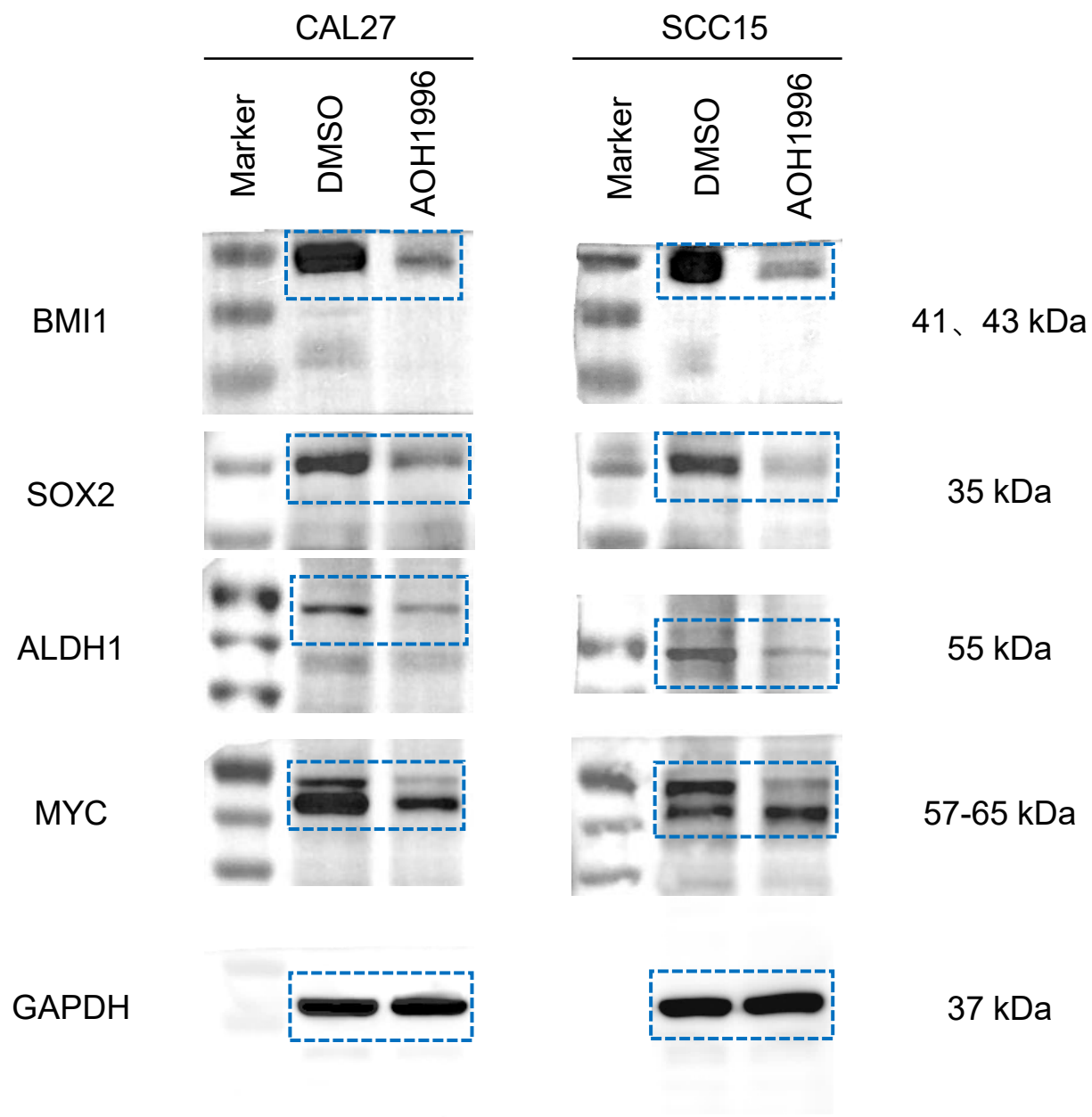

Figure 6B

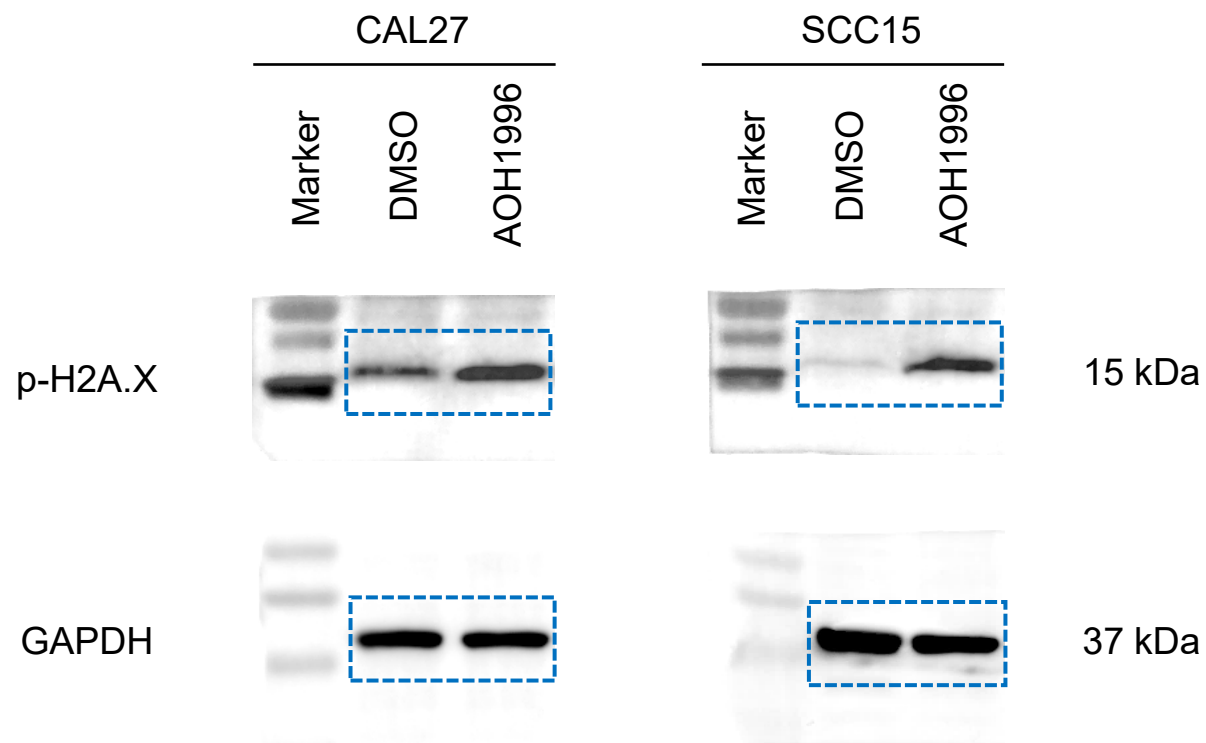

Figure 6F

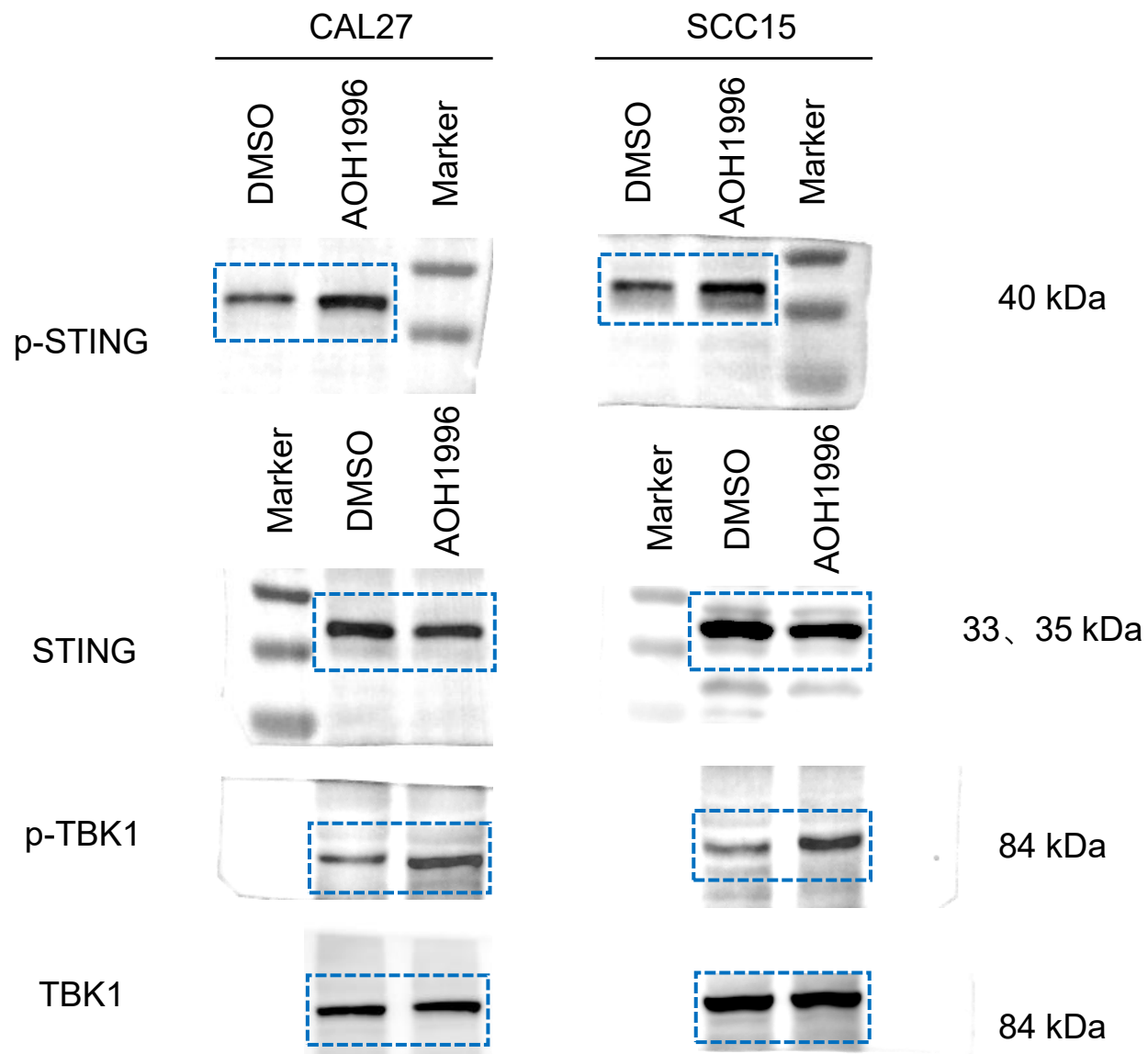

Figure 6F

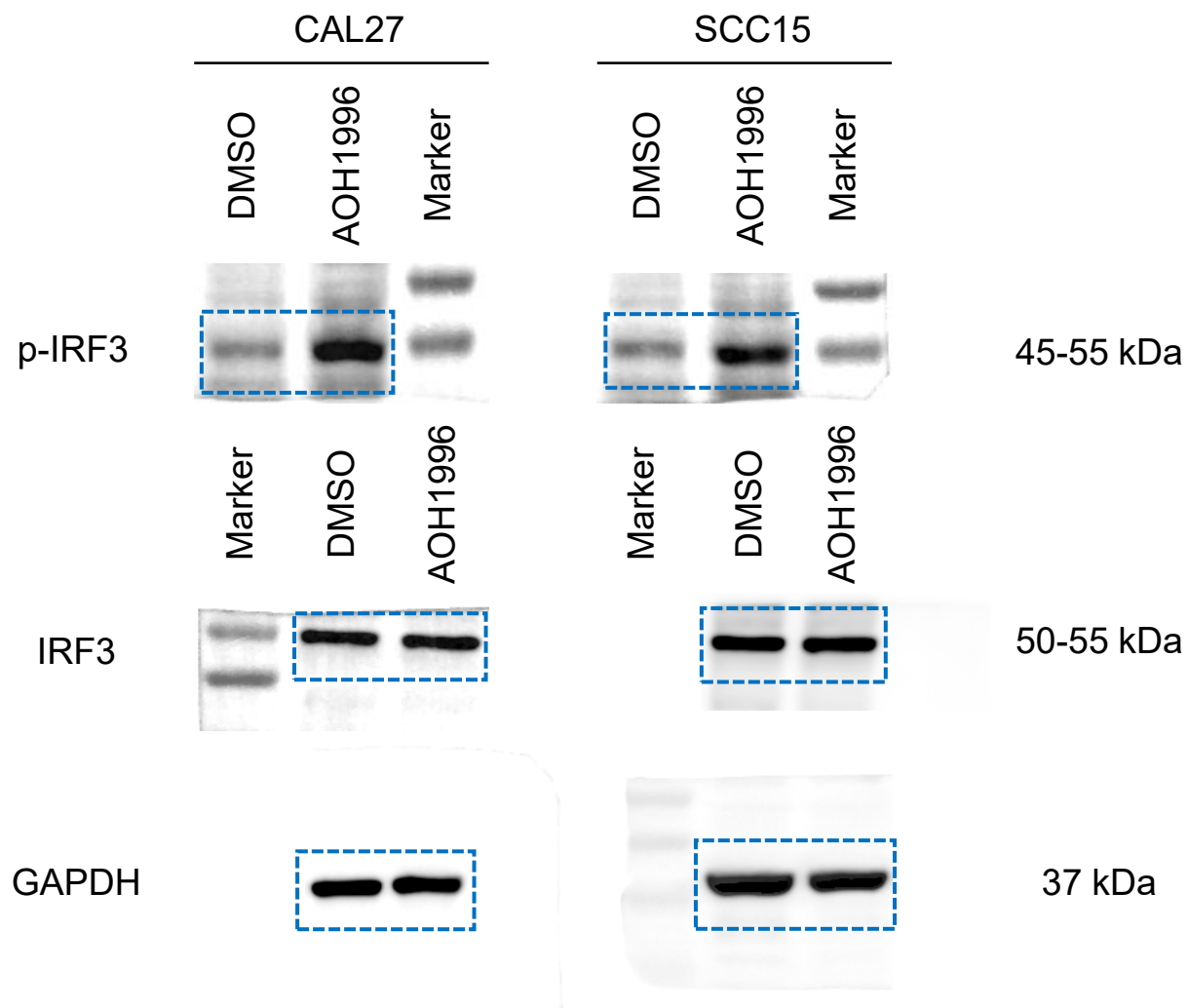

Supplement: Supplementary file 2 — Supplementary Material 2 [file 13287_2025_4607_MOESM2_ESM.pdf]
